# Primary Intranodal Hemangioma of the Axilla in a 42-Year-Old Woman: A Case Report and Review of the Literature

**DOI:** 10.7759/cureus.115017

**Published:** 2026-08-23

**Authors:** Kaushik Saha, Fatemah Modhaffar, Hesham E Kantoush, Razan A Bashir

**Affiliations:** 1 Pathology, Tata Main Hospital, Jamshedpur, IND; 2 Histopathology, Ahmadi Hospital-Kuwait Oil Company, Ahmadi, KWT; 3 Surgery, Ahmadi Hospital-Kuwait Oil Company, Ahmadi, KWT

**Keywords:** axillary lymphadenectomy, axillary lymphadenopathy, immunohistochemistry, intranodal hemangioma, vascular tumor

## Abstract

Primary intranodal hemangioma (PIH) is an exceptionally rare benign vascular neoplasm. Owing to its clinical and radiological resemblance to metastatic lymphadenopathy, it poses a diagnostic challenge, particularly when encountered in the axillary region.

We report a 42-year-old lactating woman who presented with a palpable, enlarging left axillary mass. Mammography and ultrasound identified a heterogeneous complex lesion in the left axilla. Initial ultrasound-guided core needle biopsy revealed thin-walled vascular channels consistent with hemangioma. Subsequent excision of the axillary lymph nodes demonstrated a well-circumscribed medullary vascular proliferation composed of capillary clusters with characteristic hobnail endothelial morphology. Immunohistochemistry showed diffuse positivity for CD31 and CD34, with negativity for HHV-8, confirming the diagnosis.

To the best of our knowledge, this is the first reported case of PIH in Kuwait, with fewer than 50 cases documented in the world literature. Unlike the majority of published cases, which are incidental findings during oncological staging, this lesion was detected in a patient with no prior malignancy.

Intranodal hemangioma should be considered in the differential diagnosis of solitary axillary masses, including in otherwise low-risk patients. Definitive diagnosis rests on histopathological and immunohistochemical evaluation, which is indispensable for excluding aggressive vascular neoplasms and ensuring conservative management.

## Introduction

Hemangiomas are among the most common benign vascular neoplasms, typically arising in cutaneous, mucosal, and visceral sites such as the liver and kidneys. Although predominantly encountered in the pediatric population, adult-onset hemangiomas are well recognized and exhibit a marked female preponderance (with a ratio of 6.3:1) across all clinical subtypes [1]. In contrast, primary vascular tumors arising within the lymph node parenchyma are extraordinarily rare. To date, fewer than 50 cases of primary intranodal hemangioma (PIH) have been reported in the indexed literature, underscoring the diagnostic rarity of this entity [2].

The diagnostic difficulty of PIH stems primarily from its clinical mimicry of malignant lymphadenopathy or nodal metastatic disease. In the axillary region -- a common site of presentation -- this distinction carries particularly significant management implications, given the proximity to breast tissue and the high index of suspicion for malignancy in this location. We report a case of PIH presenting as a symptomatic axillary mass in a 42-year-old woman, discuss its distinctive clinicopathological features, and review the pertinent literature.

## Case presentation

A 42-year-old woman presented with a progressively enlarging left axillary mass for the last two years. She had no significant past medical or surgical history. Her family history was notable for breast carcinoma in a maternal aunt, prompting further evaluation.

In addition, a large, round, circumscribed, heterogeneous complex mass was identified in the left axilla affecting the apical group of lymph nodes. These findings prompted an ultrasound-guided core needle biopsy of the axillary lesion. The biopsy specimen comprised a tissue core measuring 3.5 × 1.8 cm, admixed with blood clot. Microscopic examination revealed fibrocollagenous stroma containing thin-walled vascular channels lined by a single layer of flat, cytologically bland endothelial cells, with intraluminal erythrocytes. No atypical endothelial cells, papillary formations, or mitotic activity were identified. These features were interpreted as consistent with hemangioma, and excision was recommended for definitive characterization. Approximately one month later, excision of multiple left axillary lymph nodes was performed under general anesthesia, and a surgical site drain was placed in the site.

The lymphadenectomy specimen comprised fibrofatty tissue measuring 9.0 × 6.0 × 2.0 cm. Gross examination revealed an oval, hemorrhagic nodule measuring 4.5 × 2.0 × 1.3 cm with a spongy cut surface.

**Figure 1:**
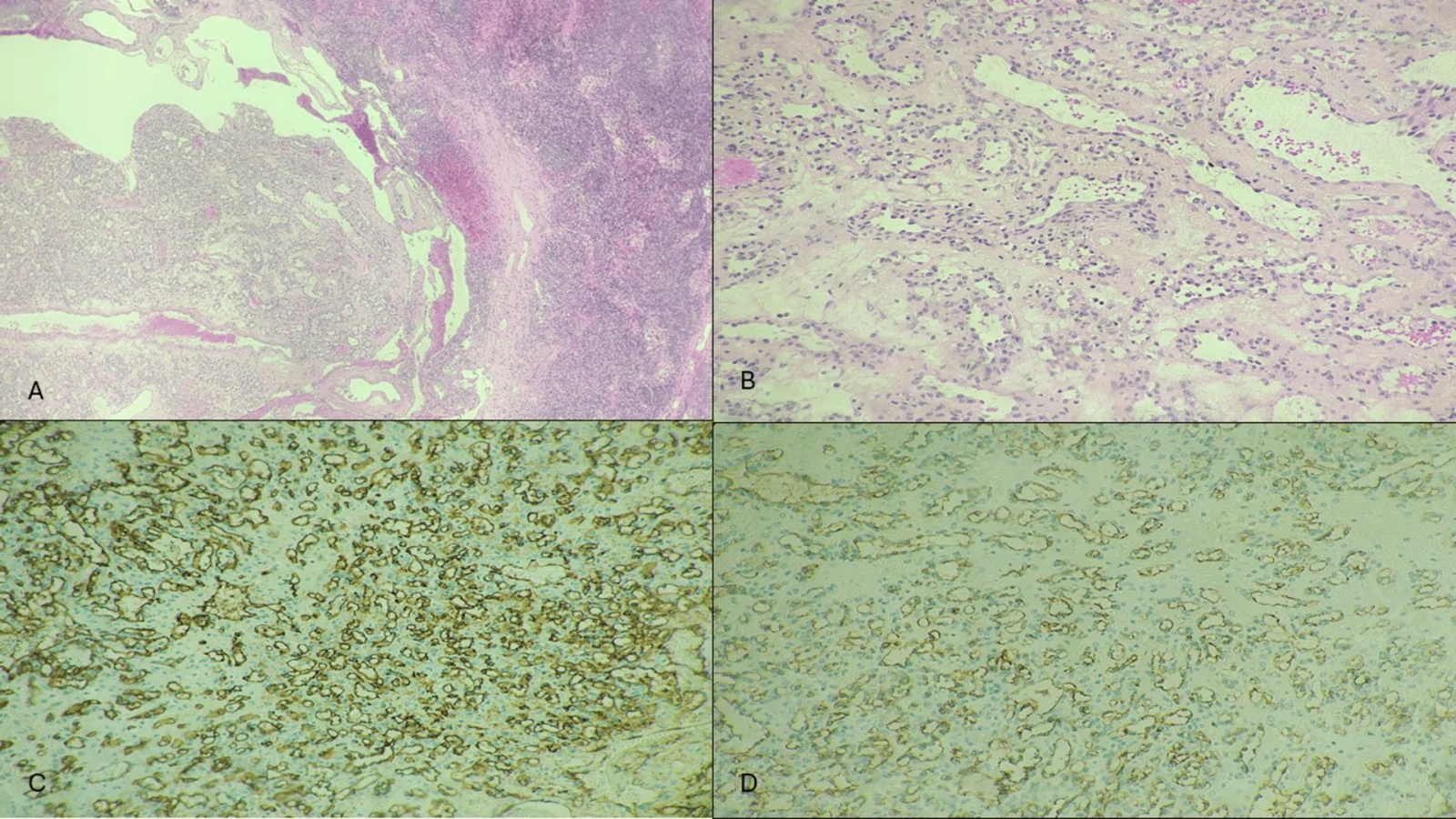
Histopathological and IHC image of the lesion (A) Low magnification of the lesion showing medullary localization of the vascular proliferation (hematoxylin and eosin, 4x). (B) Higher magnification showing slit-like capillary vascular channels with uniform endothelial cells (hematoxylin and eosin, 40x). (C) Immunohistochemical study shows diffuse strong CD34 expression in the endothelial cells (DAB, 20x). (D) Luminal surface expression of CD31 in the endothelial cells (DAB, 20x).

Histological sections showed a lymph node with preserved architecture and reactive follicular hyperplasia. Within the medulla, a well-circumscribed vascular proliferation was present, composed of lobular clusters of capillary-sized vessels with characteristic hobnail morphology of the lining endothelial cells -- nuclei bulging into the lumina without significant cytological atypia, mitotic activity, or necrosis. The surrounding lymphoid tissue showed only reactive changes, with no evidence of lymphoma, metastatic carcinoma, or other lymphoproliferative process (Figures 1A, 1B).

Immunohistochemical studies demonstrated strong, diffuse positivity of the lesional endothelial cells for CD31 and CD34 (Figures 1C, 1D). Staining for HHV-8 (latent nuclear antigen-1) was negative, effectively excluding Kaposi sarcoma (KS). Ki-67 proliferative index was low. Based on the histomorphological and immunophenotypic findings, a definitive diagnosis of PIH was established. 

After three days of the surgery, the patient was stable and afebrile, and the dressing was dry. No edema was noted in the left upper limb, with appropriate distal circulation. The patient was followed up in the surgical OPD; there was no edema of the left hand, and the left axillary scar was healthy. A follow-up ultrasound was advised, with comparison with preoperative images. 

The previous mass in the axilla was not observed in the post-operative study due to recent surgery. Multiple axillary lymph nodes were observed; some of which are prominent, the largest measuring 0.7 x 0.89 cm with a cortex thickness of 0.3 cm. The finding suggested inflammatory changes after resection.

**Figure 2:**
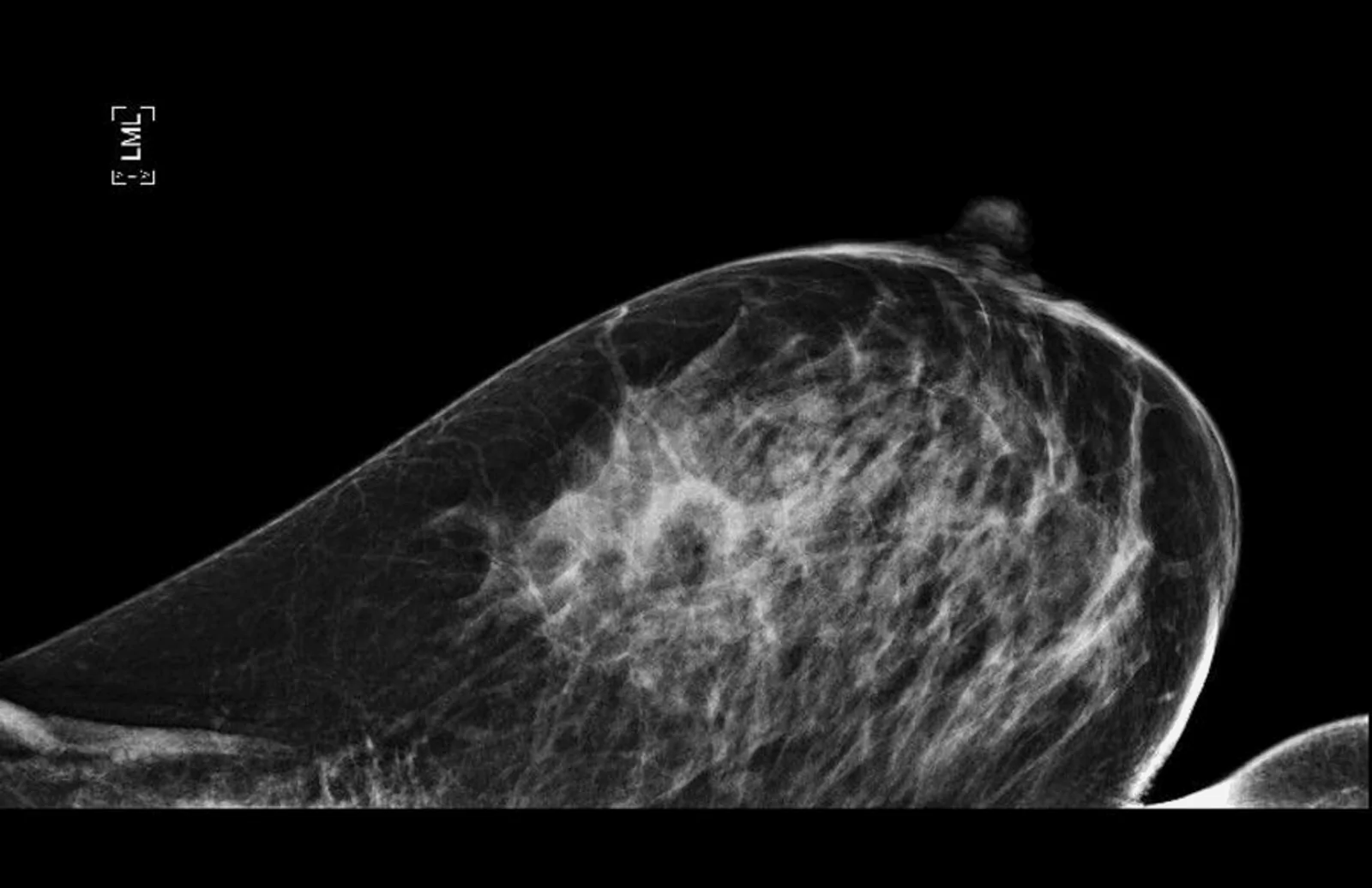
Mammography image of the left breast Mammography demonstrated a few circumscribed, high-density lesions in the upper outer quadrants of both breasts, consistent with simple cysts.

Bilateral mammography performed preoperatively demonstrated a few circumscribed, high-density lesions in the upper outer quadrants of both breasts, consistent with simple cysts (Figure 2).

Until now the patient is doing well, and there is no operative complication or recurrence.

## Discussion

Vascular proliferations within lymph nodes encompass a spectrum of entities ranging from benign reactive conditions to aggressive malignancies, including angiosarcomas. PIH occupies the far benign end of this spectrum and remains exceptionally rare; the global literature contains fewer than 50 documented cases [2]. To our knowledge, this is the first reported case of PIH from Kuwait, adding a novel geographical perspective to an already limited body of evidence.

Clinically, PIH typically presents as an asymptomatic, slowly enlarging, solitary nodal mass. Unlike most benign neoplasms, it does not regress spontaneously and may occasionally cause secondary complications, including hemorrhage, pain, or superimposed infection [3,4]. Notably, the majority of cases (approximately 55%) are discovered incidentally during lymphadenectomy or surgical staging for unrelated malignancies, particularly breast and lung carcinoma [2]. Our case deviates from this pattern: the lesion was detected in an otherwise healthy woman without any prior or concurrent oncological diagnosis, emphasizing that PIH can present de novo and should not be dismissed as a secondary finding exclusive to cancer patients.

The principal diagnostic challenge lies in distinguishing PIH from aggressive vascular neoplasms, particularly angiosarcoma and KS. This distinction is especially critical in the axilla, where the clinical suspicion for malignancy is heightened by the proximity to breast tissue. Angiosarcoma may be excluded by the absence of cytological atypia, anastomosing vascular channels, necrosis, and a high mitotic index. KS is reliably excluded by HHV-8 negativity on immunohistochemistry. CD31 and CD34 positivity supports an endothelial lineage but is non-specific; it is the combination of bland histomorphology with a negative HHV-8 and the absence of high-grade features that permits a confident diagnosis of PIH [3-5]. 

Vascular transformation of sinuses (VTS) represents an important benign differential diagnosis of PIH. VTS is a reactive, non-neoplastic vascular proliferation characterized by conversion of subcapsular and medullary sinuses into anastomosing vascular channels, often associated with fibrosis and thought to be related to lymphatic or venous outflow obstruction. In contrast to intranodal hemangioma, VTS involves and expands pre-existing nodal sinuses rather than forming a discrete circumscribed vascular mass. Furthermore, VTS lacks significant cytologic atypia and remains confined to the nodal sinusoidal compartment [6]. Epithelioid hemangioendothelioma (EHE) should also be considered in the differential diagnosis, particularly when epithelioid endothelial cells are present. Unlike benign intranodal hemangioma, EHE is a malignant endothelial neoplasm of intermediate biologic behavior with metastatic potential. Histologically, EHE is characterized by cords and nests of epithelioid endothelial cells embedded within a distinctive myxo-hyaline stroma and may demonstrate infiltrative growth. Recognition of these features, together with appropriate immunohistochemical and molecular studies, helps distinguish EHE from benign vascular lesions of lymph nodes [7]. 

The exact etiology of PIH remains incompletely understood; however, the exclusive female predisposition -- reported as 100% in the series of Sheikh and Danforth, which identified seven PIH cases among 2,461 consecutive hemangiomas examined [5] -- strongly implicates hormonal factors in its pathogenesis. We postulate that estrogen, a well-established mediator of angiogenesis and vascular endothelial growth factor upregulation, may promote the proliferation and enlargement of pre-existing or nascent nodal vascular ectasias, rendering them clinically detectable [8,9]. Future prospective work examining hormonal receptor expression in PIH specimens would be valuable in confirming this proposed mechanism.

With respect to management, PIH is a benign lesion, and its complete surgical excision is both diagnostic and curative. No adjuvant therapy is required, and long-term follow-up has not revealed recurrence in reported cases [2]. Recognition of PIH as a distinct pathological entity is therefore essential: an accurate diagnosis averts unnecessary radical surgery, adjuvant radiotherapy, or chemotherapy that might otherwise be administered under the erroneous assumption of malignancy.

## Conclusions

PIH is an exceedingly rare but clinically important entity that must be considered in the differential diagnosis of solitary axillary masses, including in patients without a prior or concurrent history of malignancy. This report, representing the inaugural description of PIH in Kuwait, underscores the necessity of excision biopsy as the gold standard for definitive diagnosis. Histological recognition of a well-circumscribed nodal vascular proliferation with hobnail endothelial morphology, supported by CD31 and CD34 positivity and HHV-8 negativity, enables confident exclusion of KS and angiosarcoma. Radiologically, there are currently no pathognomonic radiologic features that reliably distinguish PIH from malignant vascular neoplasms. Awareness of PIH as a distinct clinicopathological entity is critical to ensuring conservative management and an accurate prognosis for affected patients.
